# A multicategory logit model detecting temporal changes in antimicrobial resistance

**DOI:** 10.1371/journal.pone.0277866

**Published:** 2022-12-01

**Authors:** Marc Aerts, Kendy Tzu-yun Teng, Stijn Jaspers, Julio Alvarez Sanchez

**Affiliations:** 1 Interuniversity Institute for Biostatistics and Statistical Bioinformatics, Hasselt University, Diepenbeek, Belgium; 2 Data Science Institute, Hasselt University, Diepenbeek, Belgium; 3 VISAVET Health Surveillance Centre, Universidad Complutense, Madrid, Spain; 4 Department of Veterinary Medicine, College of Veterinary Medicine, National Chung Hsing University, Taichung City, Taiwan; 5 Department of Animal Health, Faculty of Veterinary Medicine, Universidad Complutense, Madrid, Spain; Imperial College London, UNITED KINGDOM

## Abstract

Monitoring and investigating temporal trends in antimicrobial data is a high priority for human and animal health authorities. Timely detection of temporal changes in antimicrobial resistance (AMR) can rely not only on monitoring and analyzing the proportion of resistant isolates based on the use of a clinical or epidemiological cut-off value, but also on more subtle changes and trends in the full distribution of minimum inhibitory concentration (MIC) values. The nature of the MIC distribution is categorical and ordinal (discrete). In this contribution, we developed a particular family of multicategory logit models for estimating and modelling MIC distributions over time. It allows the detection of a multitude of temporal trends in the full discrete distribution, without any assumption on the underlying continuous distribution for the MIC values. The experimental ranges of the serial dilution experiments may vary across laboratories and over time. The proposed categorical model allows to estimate the MIC distribution over the maximal range of the observed experiments, and allows the observed ranges to vary across labs and over time. The use and performance of the model is illustrated with two datasets on AMR in *Salmonella*.

## Introduction

### Challenges

The identification and understanding of temporal trends in antimicrobial data is of high importance to public health authorities (European Food Safety Authority and European Centre for Disease Prevention and Control [1]). Antimicrobial data from serial dilution experiments are registered as minimum inhibitory concentration (MIC) values, defined as the minimal concentration of an antimicrobial substance that inhibits the growth of a pathogen. A higher MIC value of a bacterium for an antimicrobial indicates a higher resistance to this antimicrobial and, when it increases beyond a certain threshold (clinical breakpoints) it may indicate a public health or animal health issue since treatment with this antimicrobial may be no longer effective [2].

Each observed discrete MIC value (often transformed on base 2 logarithmic scale) represents an interval of an unobserved true MIC value. The observed discrete MIC data typically refer to unobserved (latent) MIC values within particular intervals on continuous exponentiated scale (base 2). Thus, in statistical terms, MIC data are censored: left-, interval-, and right-censored. For example, if the range is specified as ≤ −2, −1, 0, 1, 2, 3, > 3 for a particular assay, the value ≤ −2 refers to the interval [0, 0.25] (left-censored), a value −1 ≤ *j* ≤ 3 corresponds to an unobserved “true” value in the interval (2^*j*−1^, 2^*j*^] (interval-censored, e.g (0.25, 0.5] for *j* = −1), and the value > 3 refers to the interval (8, + ∞) (right-censored). The typical range of observed (discrete) values differs with the antimicrobial type. A further complication is that the observed range is not fixed, but rather assay dependent. As a dataset typically includes data from different assays, the ranges may differ, and a particular value *j* may need to be considered left/right-censored for some observations and interval-censored for others. The objective of the design of an assay is typically to set up the range such that it is centered around one particular value, the clinical breakpoint (when available) and/or the antimicrobial type specific epidemiological cut-off value (ECV or ECOFF). This cut-off value intends to identify the value separating the “wild type” subpopulation and the subpopulation with acquired or mutational (increased) resistance to the drug of interest. Using agreed criteria for the acceptance of MIC distributions, (official or tentative) ECOFFs can be then proposed by national and international agencies (e.g., Clinical and Laboratory Standards Institute (CLSI), European Committee on Antimicrobial Susceptibility Testing (EUCAST) [3].

### Existing methodology

The last 10–15 years, several statistical methods and models with different objectives have been developed to analyse MIC data. The statistical methods vary from fully nonparametric approaches, with no assumptions on the MIC distribution, over semi-parametric approaches (partly without and partly with assumptions on the MIC distribution) to fully parametric distributions with a fully specified underlying continuous MIC distribution. The more parametric approaches are in general more efficient, but are prone to misspecifications, which may lead to possible bias(es). Nonparametric approaches are more robust, but at the cost of being less efficient. Choosing between more or less parametric approaches is not only driven by opting for efficiency versus robustness, but also by the focus of the research question at hand. If deeper insight in the underlying, latent continuous MIC distribution is envisaged, or the confirmation/determination of an ECOFF is the objective, the more parametric approaches are the preferred candidates. If one aims at detecting temporal changes, all approaches are candidates, each of them having strengths and weaknesses.

The most basic approach collapses the full observed range to dichotomised resistance data (“prevalence” of resistance with an isolate considered as resistant if its MIC value is above a given threshold, typically the ECOFF). Approaches include the Cochran-Armitage trend test or logistic regression trend models [6]. The advantage is the simplicity of the binary outcome, avoiding any issue with the unknown continuous MIC distribution, any issue concerning censoring and varying ranges. In that sense it is a nonparametric, categorical approach. Furthermore, it allows to easily apply more complex time trend models, including fractional polynomials, splines, seasonal effects etc., as well as the inclusion of other complexities in the data, such as hierarchical structures (MIC data correlated in one or more intermediate clusters, such as labs or countries), using generalized linear mixed models and GEE (generalized estimating equations) methodology [4, 5].

But there are some major disadvantages as well. There is of course the dependence on the choice of the ECOFF, especially in case there is no generally accepted unique ECOFF. Next, not all available information is being used in the trend analyses, and more detailed and subtle information about any time trends may be lost by collapsing the MIC data. In fact, for a particular ECOFF, no significant changes in the “resistance prevalence” might be detected, whereas there may be significant and interesting time related changes in the full categorical MIC distribution (and in the underlying unobserved continuous MIC distribution). Also in case the basic logistic regression approach does detect any major time trend, it is not unimportant to have more complete insights in changes in the full MIC distribution.

Using a baseline multicategory logit model is a natural extension of the binary logistic regression model, by using the full discrete/categorical range of the MIC data, and avoiding the use of a cut-off value. While such multicategory logit models share the advantages of classical logistic regression, model building and interpretation is somewhat more complex, as more than one model is involved (the number of models equals the number of MIC values minus one). As illustrated and discussed in Aerts *et al*. [6], the strength is the use of the full scale of MIC values, and being nonparametric, as there is no need to specify the underlying continuous MIC distribution. But, the baseline logit model ignores the ordinality of the MIC values, implying a less efficient approach, whereas models for ordinal categorical data such as the popular proportional odds model (or other members of the cumulative logit models) do exploit ordinality. The family of cumulative logit models assumes however a unimodal underlying continuous MIC distribution (such as the normal or the logistic distribution, not a realistic choice for the MIC distribution). Consequently, the so-called common slope assumption of such models is violated, turning them less attractive. A further weakness is that the same range of MIC values, and thus categories, should be used. We will come back to models for ordinal categorical MIC data and varying experimental ranges in the next section.

Other approaches are defined on the unobserved continuous MIC distribution, and require the partial or full specification of this continuous MIC distribution. These methods can be considered as extensions of linear regression models with a mixture distribution for the MIC response variable. The strength of these approaches is that they go beyond the limitation of the design of serial dilution experiments (implying the discrete nature of the observed MIC distribution). The price to pay is that more complex estimation methods are required (fitting mixtures taking into account the censoring), that an appropriate continuous bi-/multinomial distribution has to be selected, or has to be assumed, implying possible misspecification and bias.

Craig [7] proposed a mixture of Gaussian distributions with resistant and non-resistant populations for the underlying MIC distribution. Jaspers *et al*. [8] developed a Bayesian approach to the semiparametric estimation of the MIC distribution, with an assumed parametric distribution for the left wild type subpopulation and a nonparametric (unspecified, flexible and data-driven) model for the right resistant subpopulation. Komárek [9] presents an R package for Bayesian estimation of mixtures allowing for selection of the number of components, dealing with the censored nature of the observed MIC data. Jaspers *et al*. [10] extended this approach to multivariate mixtures to fit the joint distribution of MIC data on two or more antimicrobials, with covariate-depending mixing weights allowing to examine time trends. Zhang *et al*. [11] developed a hierarchical Bayesian latent class normal mixture model that incorporates a linear trend for the mean log2MIC of the non-resistant population.

### Contribution and outline

In this contribution we turn back to the categorical type of models, avoiding any assumptions regarding the underlying continuous MIC distribution. Using the baseline category logit model as the starting point, we define new models for ordinal categorical data reflecting the particular properties of MIC data: the central role of the ECOFF; the censored nature of the data; potentially the varying experimental ranges; and, in case of data collected over time, the temporal trend.

The following section introduces the new family of categorical models in detail. Next, the ISU VDL data and the CIPR data are briefly described, and different members of the new family are fit to both datasets. The Akaike information criterion [12] (AIC) and Schwarz’s Bayesian information criterion [13] (BIC) model selection criteria are applied to select a final model. For both criteria, lower values indicate better fitting models. Penalizing more for complexity of a model, the BIC tends to select more parsimonious models [14]. The final model is then discussed, and its estimates are interpreted subsequently. A final discussion ends the paper.

## Materials and methods

### Minimum inhibitory concentration data

Consider MIC data *y* = 2^*j*^ (or *y* = *j* on log2-scale) within its experimental range *ℓ* ≤ *j* ≤ *u* + 1. For *ℓ* < *j* ≤ *u*, the continuous unknown MIC value takes a value in the interval (2^*j*−1^, 2^*j*^] (interval censored) The case *j* = *ℓ* stands for −∞ < *j* ≤ *ℓ*, or the continuous unknown MIC value (on the original scale) takes a value in the interval (0, 2^*ℓ*^] (left censored). And finally, *j* = *u* + 1 stands for *u* < *j* < ∞, or the continuous unknown MIC value (on original scale) takes a value in the interval (2^*u*^, ∞) (right censored).

The bounds *ℓ*, *u* may vary across observations but not in a random way; they are set by the design of the experiment. Here, the categorical MIC values *y* = 2^*j*^ or, equivalently *y* = *j* on log2-scale, is considered as a categorical random variable, and interest goes to the estimation of the probabilities

$$\begin{array}{c} P(y=j),\;\;\;L=\text{min}\{\ell \}\leq j\leq \text{max}\{u\}+1=U+1, \end{array}$$ (1)

with the convention that

$$\begin{array}{c} P(y=j)≔P(j-1<y\leq j), \end{array}$$ (2)

and

$$\begin{array}{c} P(y=L)≔P(y\leq L),\;\;P(y=U+1)≔P(y>U). \end{array}$$ (3)

It might happen that the overall bounds *L*, *U* are never observed. In case the lower bound *L* is never observed, we redefine

L=min{y},

and in case the upper bound *U* is never observed, we redefine

U=max{y}-1,

with the interpretation

P(y=L)≔P(L-1<y≤L),P(y=U+1)≔P(U<y≤U+1).

Finally, for convenience, the categories *L*, …, *U* + 1 will be relabeled as 1, …, *U* − *L*. For instance, for the CIPR data min{*ℓ*} = −7 and max{*u*} = 5.

### The epidemiological cutoff baseline logit model

The baseline logit model models the log of the odds of the probability *P*(*y* = *j*) against *P*(*y* = *baselinecategory*). The choice of the baseline or reference category is in general arbitrary, and different choices lead to equivalent models. Typical choices include, the first, the last or the most frequent category. In the context of AMR, a natural choice is the category corresponding to the ECOFF, as this ECOFF dichotomizes the MIC distribution in the wild-type part (left of ECOFF) and the non-wild type (or resistant) part (right of ECOFF).

Denoting the ECOFF category as *J*_E_, the baseline category logit model can be written as, with *x* representing a covariate of interest:

$$\begin{array}{c} \text{log}\;\frac{P(y=j|x)}{P(y=J_{\text{E}}|x)}=\alpha_{j}+f_{j}\left(x\right),\;\;\;L\leq j\leq U+1, \end{array}$$ (4)

with the constraints

$$\alpha_{J_{\text{E}}}=0,\;\;f_{J_{\text{E}}}\left(x\right)=0,\;\;f_{j}\left(0\right)=0.$$

Given a sample {(*x*_1_, *y*_1_), …, (*x*_*n*_, *y*_*n*_)}, estimates can be obtained by maximum likelihood (MLE) by maximizing the log-likelihood

$$\text{log}\;\text{L}=\sum_{i=1}^{n}\sum_{L\leq j\leq U+1}\delta_{ij}\text{log}\;P\left(y_{i}=j|x_{i}\right)$$

with *δ*_*ij*_ = 1 if *y*_*i*_ = *j*, and 0 otherwise.

In this paper, the covariate *x* of interest is time, but *x* can also represent other characteristics of interest, such as the presence of a plasmid or a genetic variant.

### Accounting for varying experimental ranges

The above definition of the baseline logit model cannot be fitted to our datasets in the application setting, as it ignores the varying experimental ranges *ℓ*, *u* and the censored nature of the data. To accommodate this, the likelihood is modified similar to the likelihood for censored continuous type of data. Consider the following cases for observation *y*_*i*_ within the range {*ℓ*_*i*_, *ℓ*_*i*_ − 1, …, *u*_*i*_ − 1, *u*_*i*_, *u*_*i*_ + 1}, its value *j*, and corresponding contribution to the log-likelihood: for *ℓ*_*i*_ < *y*_*i*_ = *j* ≤ *u*_*i*_ the contribution is *P*(*y*_*i*_ = *j*|*x*_*i*_); for *y*_*i*_ = *ℓ*_*i*_ it is $P\left(L\leq y_{i}\leq \ell_{i}|x_{i}\right)=\sum_{j=L}^{\ell_{i}}P\left(y_{i}=j|x_{i}\right)$; and for *y*_*i*_ = *u*_*i*_ + 1 the contribution is $P\left(u_{i}<y_{i}\leq U+1|x_{i}\right)=\sum_{j=u_{i}+1}^{U+1}P\left(y_{i}=j|x_{i}\right)$. This results in the log-likelihood expression $\text{log}\;\text{L}=\sum_{i=1}^{n}\text{log}\;{\text{L}}_{i}$, with log *L*_*i*_ given by

$$\begin{array}{c} \delta_{i\ell_{i}}\text{log}\;\left(\sum_{j=L}^{\ell_{i}}P\left(y_{i}=j|x_{i}\right)\right)+\sum_{\ell_{i}<j\leq u_{i}}\delta_{ij}\text{log}\;P\left(y_{i}=j|x_{i}\right)+\delta_{i,u_{i}+1}\text{log}\;\left(\sum_{j=u_{i}+1}^{U+1}P\left(y_{i}=j|x_{i}\right)\right). \end{array}$$ (5)

### Accounting for the ordinality of the MIC categories

The model as defined above does not yet exploit the ordinality of the MIC categories *L* ≤ *j* ≤ *U* + 1. Exploiting ordinality typically allows building more parsimonious and more powerful models, with less association parameters, being homogeneous in one or more directions. As such the baseline model is not the optimal choice, but it can be fitted to ordinal categorical response data and the model can be modified to reflect the ordinal nature of the MIC data. Reconsider the model formula (4). The basic baseline logit model, with a linear effect of time, would correspond to the choice

$$\begin{array}{c} f_{j}\left(x\right)=\beta_{j}x, \end{array}$$ (6)

with the constraint that $\beta_{J_{\text{E}}}=0$. The effect of time could be modelled differently, not just linear, but in a saturated way with dummies, or with e.g. a quadratic effect next to the linear effect. But characteristic for the baseline model is that each logit $\text{log}\;\frac{P(y=j|x)}{P(y=J_{\text{E}}|x)}$ gets a different slope (all *β*_*j*_ different). Reason for this is that, for nominal data, the categories can be permuted randomly. Here, however, we have ordered categories *L* ≤ *j* ≤ *U* + 1, and we might expect a positive or negative trend over time. Intuitively, as a starting point, one might expect the following effect of time on the MIC distribution:

1. a shift from the susceptible part to the resistant part, or, with *x* denoting time
  $$\text{log}\;\frac{P(\text{isolate}\;\text{is}\;\text{resistant}|x)}{P(\text{isolate}\;\text{is}\;\text{susceptible}|x)}=\text{log}\;\frac{P(y>J_{\text{E}}|x)}{P(y\leq J_{\text{E}}|x)}\;\;\text{increases}\;\text{as}\;\text{a}\;\text{function}\;\text{of}\;\text{time}\;x,$$
2. the probabilities related to the susceptible part do not change with time (wild-type distribution remaining unchanged), so for *j*_*s*_ ≤ *J*_E_,
  $$P(y=j_{s}|y\leq J_{\text{E}},x)\;\;\text{do}\;\text{not}\;\text{depend}\;\text{on}\;\text{time}\;x,$$
3. the probabilities related to the resistant part do change with time (moving to the right), so for *j*_*r*_ > *J*_E_,
  $$\text{log}\frac{P(y=j_{r}+1|y>J_{\text{E}},x)}{P(y=j_{r}|y>J_{\text{E}},x)}\;\;\text{increase}\;\text{as}\;\text{a}\;\text{function}\;\text{of}\;\text{time}\;x.$$

The challenge is to identify one or more models that fit well to the data, based on parsimonious parameterizations, which reflect (fully or partly) the above preliminary expectations. In addition, models not consistent with these expectations have to be explored as well.

### The family of models

A family of models representing particular trends and recognizing the central role of the ECOFF is defined by (subscripts *s* and *r* referring to the categories to the left (susceptible) and to the right (resistant) of the ECOFF):

$$\begin{array}{c} f_{j}\left(x\right)=\{\begin{array}{cc} f_{s}\left(j-J_{\text{E}}\right)\beta_{s}x & \text{for}\;\;\;L\leq j\leq J_{\text{E}},\\ f_{r}\left(j-J_{\text{E}}\right)\beta_{r}x & \text{for}\;\;\;J_{\text{E}}\leq j\leq U+1, \end{array} \end{array}$$ (7)

with *f*_*s*_, *f*_*r*_ “score” functions reflecting the ordinal structure with the constraint that *f*_*s*_(0) = *f*_*r*_(0) = 0 (implying that $f_{J_{\text{E}}}\left(x\right)=0$). This model contains two covariate-effect parameter *β*_*s*_ and *β*_*r*_ (possibly identical), acting with multiplicative factors *f*_*s*_(*j* − *J*_E_), *f*_*r*_(*j* − *J*_E_) across all logits, in a possibly different fashion left and right of the ECOFF. The linear effects *β*_*s*_*x*, *β*_*r*_*x* could be generalized to multiple linear effects with several covariates or to other particular effect functions *g*_*s*_(*x*), *g*_*r*_(*x*) (e.g. including a quadratic effect, a fractional polynomial or a periodic function).

**Fig 1:**
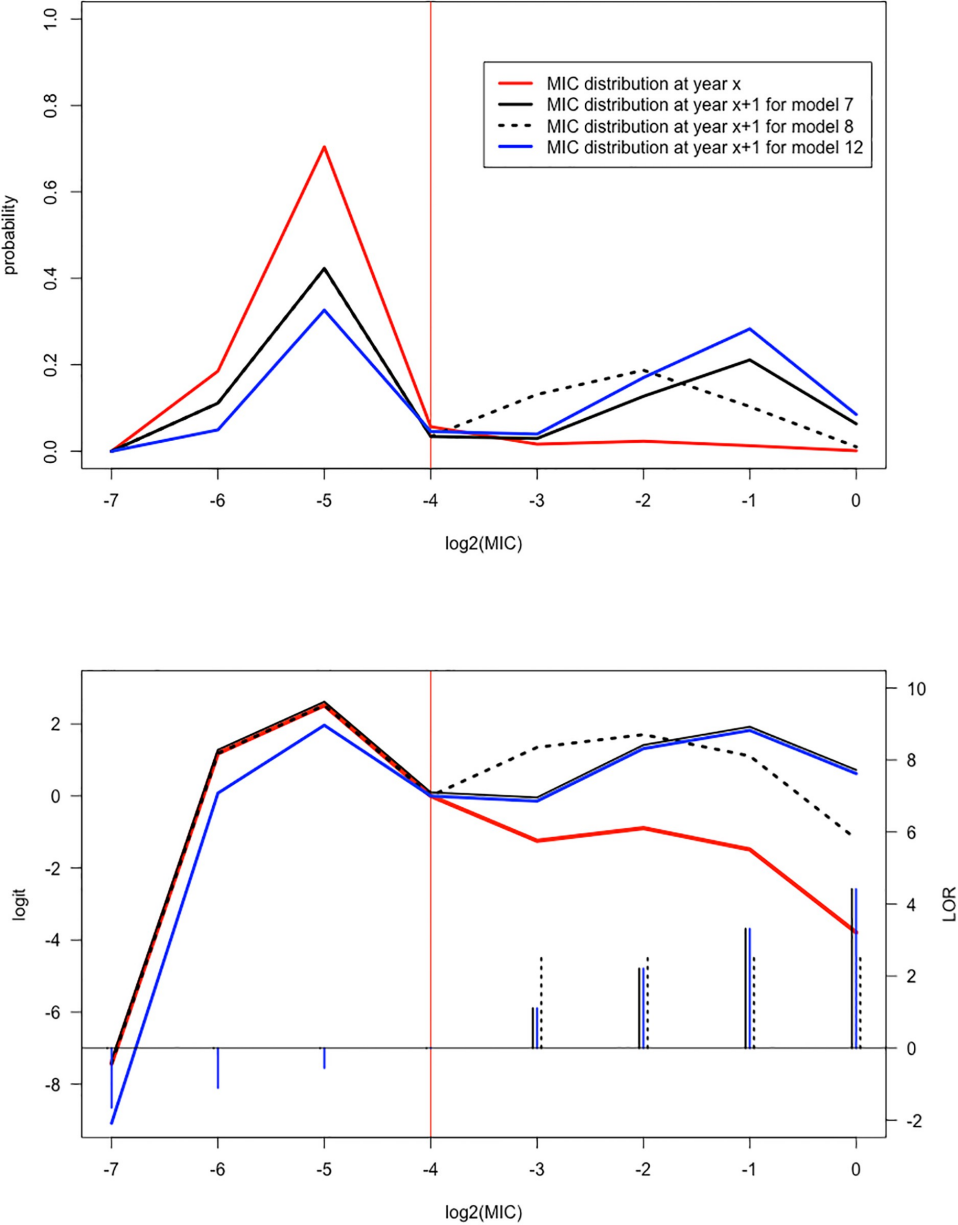
The family of models. Illustration of particular models using the identity, sign and null function, with reference to the setting of the CIPR data analysis (Table 2). The red curve refers to the MIC distribution at year *x*, the other curves refer to the distribution at year *x* + 1, according to different models: model 7 (solid, black) is the model with *f*_*s*_(*x*) = 0 and *f*_*r*_(*x*) = *x* with *β*_*r*_ > 0; model 8 (dashed, black) is the model with *f*_*s*_(*x*) = 0 and *f*_*r*_(*x*) = sign(*x*) with *β*_*r*_ > 0; model 12 (solid, blue) is the model with *f*_*s*_(*x*) = *x* with *β*_*s*_ > 0, and *f*_*r*_(*x*) = *x* with *β*_*r*_ > 0. The vertical red line refers to the ECOFF category. The upper panel shows the MIC distribution *P*(*y* = *j*), *j* = −7, …, 0 (corresponding to MIC values 2^j^). The lower panel shows the logits $\text{log}\frac{P(y=j)}{P(y=J_{\text{E}})}$. The short vertical lines at the horizontal axis in the lower panel show how these logits have changed after one year (LOR’s) according to model 7, model 8 and model 12, in their respective line types and colors.

Different choices for *f*_*s*_, *f*_*r*_ imply different models. Of particular interest are (suppressing the subscripts *s* and *r*) *f*(*x*) = *x* (identity function), *f*(*x*) = sign(*x*) (sign function), *f*(*x*) = 0 (null function, equivalent to putting the corresponding parameter *β* to 0) or other functions such as those reflecting particular scores (e.g. distances between midpoints in case the categories are not equidistant). Fig 1 demonstrates the time effect according to some of the models that play a main role in the analysis of the CIPR data analysis (Table 2). The red curves show the distribution at a particular year *x*: the MIC distribution *P*(*y* = *j*) (upper panel), and the logit $\text{log}(\frac{P(y=j)}{P(y=J_{\text{E}})})$ in the lower panel (with the ECOFF category indicated by the red vertical line). The other curves show the changes one year later, according to three models. The particular values assigned to the parameter of the models were inspired, though not equal by those of the CIPR data analysis (the effects were taken larger to show them more clearly).

Model 7 with *f*_*s*_(*x*) = 0, *f*_*r*_(*x*) = *x* (solid black) corresponds to no changes over time in the logit $\text{log}(\frac{P(y=j_{s})}{P(y=J_{\text{E}})})$ in the susceptible component of the distribution (left of the ECOFF, red and solid black curves are identical in the lower panel) and a positive effect on the resistant component (right of the ECOFF, black curve lays above the red curve in the lower panel). The log odds ratios (LOR) (*j* − *J*_E_) *β*_*r*_ show the increase over time *x* + 1 (one year later), with a linear increase of this effect over *j* − *J*_E_ = 1, 2, 3, 4. This latter linear increase over *j* (with values 1.10,2.21,3.31,4.41) is shown by the short black solid vertical lines at the horizontal axis in the lower panel of Fig 1 (with scale indicated in the right vertical axis). The corresponding MIC distribution for model 7 one year later is shown in the above panel (solid black). It shows a pronounced increase of probabilities for the larger categories in the resistant component. Model 8 (dashed black curves) is similar to model 7 (no changes in the logits at the susceptible side), but the increase, now equal to LOR *β*_*r*_, is constant over *j*. The constant increase 2.59 is shown by the short black dashed vertical lines at the horizontal axis in the lower panel. Model 12 is also similar to model 7, but at the right resistant side. This model however represents a decrease at the susceptible side as well, expressed by the LOR’s (*j* − *J*_E_)*β*_*s*_ with now *j* − *J*_E_ = −1, −2, −3. This latter linear decrease over *j* (with values -1.65,-1.10,-0.55) is shown by the short blue solid vertical lines at the horizontal axis in the lower panel. How such changes in the logits reflect changes in the MIC distribution is shown in the upper panel. Note that if more probability is assigned to the resistant component on the right, less probability is automatically assigned to the left susceptible component (as observable in the upper panel of Fig 1).

Model (7) implies particular odds ratio interpretations of the effect parameters *β*_*s*_, *β*_*r*_, for different 2 × 2 tables, as indicated by Table 1. Starting with the baseline odds, we have the relation between the odds corresponding with a unit increase in time *x*:

$$\frac{P(y=j|x+1)}{P(y=J_{\text{E}}|x+1)}={\text{OR}}_{j,J_{\text{E}}}\frac{P(y=j|x)}{P(y=J_{\text{E}}|x)}$$

with

$${\text{OR}}_{j,J_{\text{E}}}=\{\begin{array}{cc} \text{exp}\{f_{s}\left(j-J_{\text{E}}\right)\beta_{s}\} & \text{for}\;\;\;L\leq j\leq J_{\text{E}},\\ \text{exp}\{f_{r}\left(j-J_{\text{E}}\right)\beta_{r}\} & \text{for}\;\;\;J_{\text{E}}\leq j\leq U+1. \end{array}$$

Focusing to the left side of the ECOFF (*j* < *J*_E_), *f*_*s*_ ≡ 0 refers to no effect of *x* on the susceptible component; the sign function implies a single OR for all susceptible categories, and the identity function implies a muliplicative factor for each step further apart from the ECOFF category. Similar interpretations hold for the baseline odds with *j* > *J*_E_.

**Table 1:** The 2 × 2 tables of interest, to the left and to the right of the ECOFF, with notation *π*_*j*_(*x*) = *P*(*y* = *j*|*x*).

	*j*_*s*_ − 1	*j*_*s*_	*j*_E_ − 1	*j*_E_	*j*_E_ + 1	*j*_*r*_	*j*_*r*_ + 1
***x***	$\pi_{j_{s}-1}\left(x\right)$	$\pi_{j_{s}}\left(x\right)$	$\pi_{J_{\text{E}}-1}\left(x\right)$	$\pi_{,J_{\text{E}}}\left(x\right)$	$\pi_{J_{\text{E}}+1}\left(x\right)$	$\pi_{j_{r}}\left(x\right)$	$\pi_{j_{r}+1}\left(x\right)$
***x* + 1**	$\pi_{j_{s}-1}$ (*x* + 1)	$\pi_{j_{s}}\left(x+1\right)$	$\pi_{J_{\text{E}}-1}\left(x+1\right)$	$\pi_{J_{\text{E}}}\left(x+1\right)$	$\pi_{J_{\text{E}}+1}\left(x+1\right)$	$\pi_{j_{r}}\left(x+1\right)$	$\pi_{j_{r}+1}$ (*x* + 1)

Next, consider adjacent odds, to the right of the ECOFF *J*_E_ < *j*_*r*_ < *j*_*r*_ + 1 ≤ *U* + 1

$$\frac{P(y=j_{r}+1|x+1)}{P(y=j_{r}|x+1)}={\text{OR}}_{j_{r}+1,j_{r}}\frac{P(y=j_{r}+1|x)}{P(y=j_{r}|x)}$$

with

$${\text{OR}}_{j_{r}+1,j_{r}}=\text{exp}\left\{\left[f_{r}\left(j_{r}+1-J_{\text{E}}\right)-f_{r}\left(j_{r}-J_{\text{E}}\right)\right]\beta_{r}\right\}.$$

For *f*_*r*_ ≡ 0, we obtain no effect of *x* as ${\text{OR}}_{j_{r},j_{r}+1}=1$. The sign function implies ${\text{OR}}_{j_{r},j_{r}+1}=1$ as well. And the identity function leads to ${\text{OR}}_{j_{r},j_{r}+1}=e^{\beta_{r}}$. Note that these ORs are interpretable as conditional ORs, given that *y* > *J*_E_. Similar considerations and interpretations hold for conditioning on the left side *L* ≤ *j*_*s*_ < *j*_*s*_ + 1 < *J*_*E*_. One can also contrast two categories at both sides of the ECOFF *j*_*s*_ < *J*_*E*_ < *j*_*r*_:

$$\frac{P(y=j_{r}|x+1)}{P(y=j_{s}|x+1)}={\text{OR}}_{j_{r},j_{s}}\frac{P(y=j_{r}|x)}{P(y=j_{s}|x)}$$

with

$${\text{OR}}_{j_{r},j_{s}}=\text{exp}\left\{f_{r}\left(j_{r}-J_{\text{E}}\right)\beta_{r}-f_{s}\left(j_{s}-J_{\text{E}}\right)\beta_{s}\right\}.$$

Finally, model (7) implies the following odds contrasting the probabilities for resistance against susceptibility:

$$\frac{P(y>J_{\text{E}}|x)}{P(y\leq J_{\text{E}}|x)}=\frac{\sum_{j_{r}>J_{\text{E}}}e^{\alpha_{j_{r}}+f_{r}\left(j_{r}-J_{\text{E}}\right)\beta_{r}x}}{\sum_{j_{s}\leq J_{\text{E}}}e^{\alpha_{j_{s}}+f_{r}\left(j_{s}-J_{\text{E}}\right)\beta_{s}x}},$$

which looks rather complicated. But, for the case both functions *f*_*s*_ and *f*_*r*_ are the sign function, we get

$$\begin{array}{c} \frac{P(\text{isolate}\;\text{is}\;\text{resistant}|x)}{P(\text{isolate}\;\text{is}\;\text{susceptible}|x)}=\frac{P(y>J_{\text{E}}|x)}{P(y\leq J_{\text{E}}|x)}=Ce^{(\beta_{s}+\beta_{r})x}, \end{array}$$ (8)

for some constant *C*. Expression (8) is just a basic linear logistic regression model

$$\text{log}\frac{P(y>J_{\text{E}}|x)}{P(y\leq J_{\text{E}}|x)}=\tilde{\alpha }+\tilde{\beta }x,$$

with

$$\tilde{\alpha }=\{\text{log}\frac{\sum_{j>J_{\text{E}}}\alpha_{j}}{\sum_{j\leq J_{\text{E}}}\alpha_{j}}\},\;\;\tilde{\beta }=\beta_{s}+\beta_{r}.$$

If one of the functions *f*_*s*_ and *f*_*r*_ is the zero-function, this logistic regression model still holds but with slope *β*_*r*_ and *β*_*s*_ respectively.

Let us reconsider the intuitively expected time effects on the susceptible and resistant subpopulations, being a model reflecting the properties i)-iii). Consider a model with *f*_*s*_ ≡ 0, implying no effect of *x* on the susceptible subpopulation, as

$${\text{OR}}_{j_{s},j_{s}+1}={\text{OR}}_{j_{s},J_{\text{E}}}=1,\;\;L\leq j_{s}<j_{s}+1\leq J_{\text{E}}.$$

With *f*_*s*_(*x*) = sign(*x*) and *β*_*s*_ > 0:

$${\text{OR}}_{j_{s},j_{s}+1}=1,\;\;L\leq j_{s}<j_{s}+1<J_{\text{E}},$$

but

$${\text{OR}}_{j_{s},J_{\text{E}}}=e^{-\beta_{s}}<1,\;\;L\leq j_{s}<j_{s}+1\leq J_{\text{E}},$$

indicating there is a “negative” effect, reflecting a shift of higher probability to the ECOFF category *J*_E_.

For the resistant part, consider a model with *f*_*r*_ the sign function or the linear function with a slope *β*_*r*_ > 0. Both reflect a shift to more probability to the “higher” categories of the resistant subpopulation. For the linear function *f*_*r*_(*x*) = *x* this implies that, with *β*_*r*_ > 0,

$${\text{OR}}_{j_{r},j_{r}+1}=e^{\beta_{r}}>1,\;\;J_{\text{E}}\leq j_{r}<j_{r}+1<U+1,$$

and

$${\text{OR}}_{j_{r},J_{\text{E}}}=e^{\left(j_{r}-J_{\text{E}}\right)\beta_{r}}>1,\;\;J_{\text{E}}\leq j_{r}<j_{r}+1<U+1,$$

reflecting a gradual shift to higher MIC values. In combination with *f*_*s*_ the sign function, it holds that

$${\text{OR}}_{j_{r},j_{s}}=e^{\left(j_{r}-J_{\text{E}}\right)\beta_{r}+\beta_{s}}>1,\;\;j_{s}<J_{E}<j_{r}.$$

For *f*_*r*_(*x*) = *x* and *f*_*s*_(*x*) = sign(*x*) we have

$$\text{log}\frac{P(\text{isolate}\;\text{is}\;\text{resistant}|x)}{P(\text{isolate}\;\text{is}\;\text{susceptible}|x)}=\tilde{\alpha }+\beta_{s}x+\text{log}\left(\sum_{j_{r}>J_{\text{E}}}e^{\alpha_{j_{r}}+\left(j_{r}-J_{\text{E}}\right)\beta_{r}x}\right)$$

which is increasing as a function of *x*. In conclusion, intuitively, the model with *f*_*r*_(*x*) = *x* and *f*_*s*_(*x*) = sign(*x*) (or *f*_*s*_(*x*) = 0) is expected to be very plausible.

## Application to real datasets

Two datasets are analysed: the ISU VDL data with identical experimental ranges across time; the CIPR data with varying experimental ranges.

R [15] was used for coding the models and analyzing the data. R scripts are available from the corresponding author on request.

### The CIPR data

The CIPR dataset was derived from the annual antimicrobial susceptibility testing results of *Salmonella* isolates from pigs, collected through a national antimicrobial resistance monitoring program in Spain in 2002–2013. Briefly, samples of the content of caecum from pigs collected at the abattoir representing independent epidemiological units (i.e., farms) are processed for the bacteriological culture of *Salmonella*. More details on the sampling strategy of the isolates can be found in Teng *et al*. [16]. Isolates retrieved are then subjected to antimicrobial susceptibility testing using the broth microdilution method. Here we focus on the MIC data generated for the antimicrobial ciprofloxacin (CIPR).

**Fig 2:**
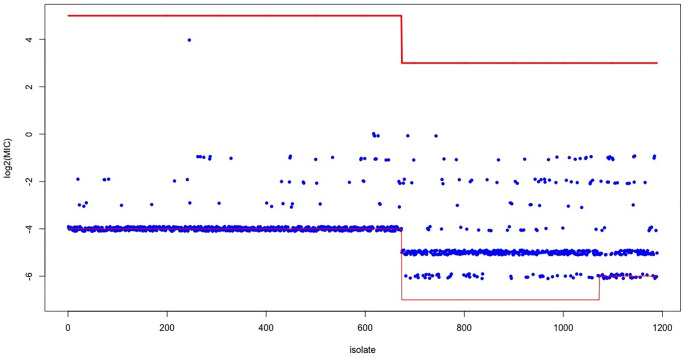
The CIPR data. Jittered MIC values in blue; the red lines show the experimental ranges.

Fig 2 shows the varying experimental ranges together with the data of 1189 isolates tested during the 2002–2013 period. The horizontal lines (in red) represent the lower and upper experimental range for groups of isolates and the blue jittered points are the observed MIC values (on log2 scale). The ECOFF equals -4. The single outlying MIC value of log2(MIC) = 4 was excluded, resulting in a total of 1188 observations. The upper part of Table 5 shows the observed counts with, in the right margin, the sample sizes varying from 40 to 211 over the 12-year period.

The full range across all isolates runs from—7 up to 5: (-4,5) in the period 2002–2007, (-7,3) in the period 2008–2011, and (-6,3) in the priod 2012–2013. A majority of observed MIC values (55%) equals the lower experimental bound (36 MIC values are equal to the lower bound -6 and 618 MIC values are equal to the ECOFF, being the lower bound -4 in the period 2002–2007, see also Fig 2 and Table 5. So the majority of MIC values are left censored. None of the MIC values exceeds the upper bound (being right-censored). Note that depending on the particular year, a value of -6 needs to be interpreted differently: for 2008 the discrete value -6 refers to the interval (-7,-6] whereas for 2012 it refers to the interval (−∞, −6].

**Fig 3:**
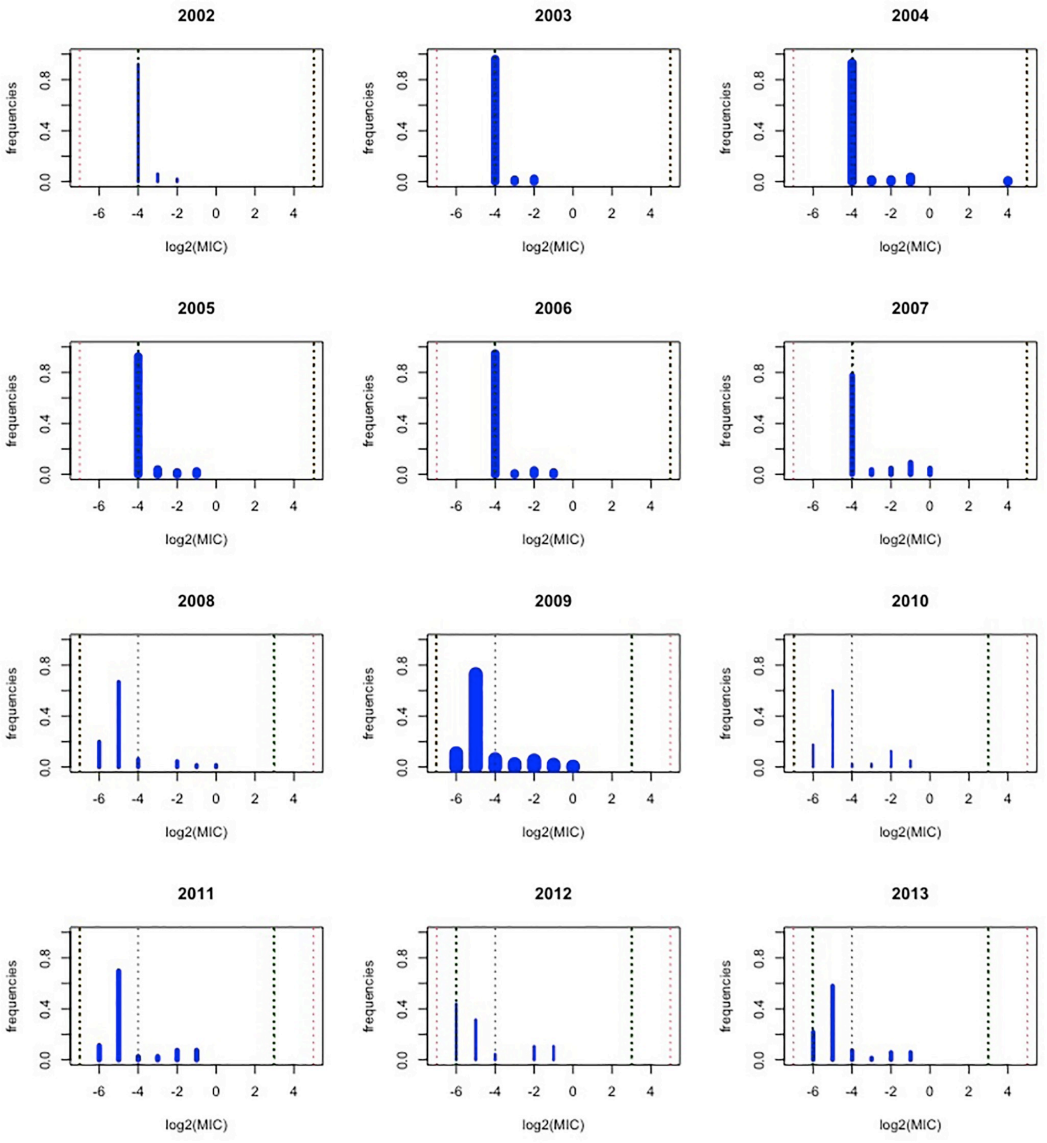
Barplots of the CIPR data. Barplots with observed relative frequencies for each year in the period 2002–2013, with barwidth proportional to the sample size of the respective year. The dotted vertical lines indicate: the smallest lower bound -7 and the highest upper bound 5 across all years (in red); the lower and upper bound of the experiments of the corresponding year (in green), the ECOFF (in black).

Fig 3 shows the observed categorical MIC distribution for each year, with the width of the bars proportional to the number of isolates tested in the corresponding year. Note that this figure is misleading. For instance, the MIC distribution for 2002 might suggest the value -4 (the ECOFF!) is most frequent, but actually this bar stands for all values ≤ −4. So, the analysis has to take this into account, and such an extreme situation might jeopardize the use of methods modelling the underlying continuous MIC distribution.

#### Standard multicategory logit models

Here we take the MIC values (categories) as they are, not correcting for the varying experimental ranges, implying that the majority of left-censored values are treated incorrectly. The upper part of Table 2 shows AIC and BIC values of naive standard multicategory logit models, with no or a linear effect of time. As there are 7 possible categories (corresponding to the observed values -6 to 0), there are 6 multicategory logits, and hence 6 intercepts and, additionally, 0, 1 common or 6 different slopes, depending on the model. This upper part of Table 2 shows that the proportional odds and the adjacent logit model (with equal slopes) do not fit well, as compared to the baseline logit model. The upper part of S1 Table shows the estimated probabilities on the MIC categories over the period 2002–2013, based on the standard baseline logit model, not accounting for the left censoring, and not exploiting the ordinal nature of the MIC distribution. The fit is to a large extent driven by the experimental lower limits. Moreover, this model implies an unlikely shift to the susceptible part of the distribution (left of ECOFF).

**Table 2:** The CIPR data: Summary goodness-of-fit statistics for all fitted models.

model	-2 log-likelihood	# par	AIC	BIC
**standard only intercepts model**	2938.2	6	2950.2	2980.7
**standard baseline logit model**	1896.0	12	1920.0	1981.0
**standard proportional odds model**	2498.7	7	2512.7	2548.3
**standard adjacent logit model**	2805.7	7	2819.7	2855.2
**1: no time effect**	1710.1	7	1724.1(15)	1759.7(14)
**2: baseline logit**	1672.2	14	1700.2(1)	1771.3(15)
**3: *f*_*s*_(*x*) = *f*_*r*_(*x*) = *x*, *β*_*s*_ = *β*_*r*_**	1693.5	8	1709.5(9)	1750.1(6)
**4:*f*_*s*_(*x*) = *f*_*r*_(*x*) = sign(*x*), *β*_*s*_ = *β*_*r*_**	1693.0	8	1709.0(8)	1749.6(5)
**5: *f*_*s*_(*x*) = *x*, *f*_*r*_(*x*) = sign(*x*), *β*_*s*_ = *β*_*r*_**	1698.2	8	1714.2(13)	1754.8(10)
**6: *f*_*s*_(*x*) = sign(*x*), *f*_*r*_(*x*) = *x*, *β*_*s*_ = *β*_*r*_**	1689.7	8	1705.7(4)	1746.4(2)
**7: *f*_*s*_(*x*) = 0, *f*_*r*_(*x*) = *x***	1689.1	8	1705.1(3)	1745.8(1)
**8: *f*_*s*_(*x*) = 0, *f*_*r*_(*x*) = sign(*x*)**	1692.6	8	1708.6(7)	1749.2(4)
**9: *f*_*s*_(*x*) = *x*, *f*_*r*_(*x*) = 0**	1702.5	8	1718.5(14)	1759.1(13)
**10: *f*_*s*_(*x*) = sign(*x*), *f*_*r*_(*x*) = 0**	1693.9	8	1709.9(10)	1750.5(7)
**11: *f*_*s*_(*x*) = sign(*x*), *f*_*r*_(*x*) = *x*, *β*_*s*_ ≠ *β*_*r*_**	1689.1	9	1707.1(5.5)	1752.8(8.5)
**12: *f*_*s*_(*x*) = *f*_*r*_(*x*) = *x*, *β*_*s*_ ≠ *β*_*r*_**	1683.6	9	1701.6(2)	1747.3(3)
**13: *f*_*s*_(*x*) = *f*_*r*_(*x*) = sign(*x*), *β*_*s*_ ≠ *β*_*r*_**	1692.6	9	1710.6(11.5)	1756.3(11.5)
**14: *f*_*s*_(*x*) = *f*_*r*_(*x*) = *x*, *β*_*s*_ ≠ *β*_*r*_, *β*_*s*_, *β*_*r*_ > 0**	1689.1	9	1707.1(5.5)	1752.8(8.5)
**15: *f*_*s*_(*x*) = *f*_*r*_(*x*) = sign(*x*), *β*_*s*_ ≠ *β*_*r*_, *β*_*s*_, *β*_*r*_ > 0**	1692.6	9	1710.6(11.5)	1756.3(11.5)

#### New family of multicategory logit models

Several members of the new family of models introduced in the materials and methods section have been fitted to the CIPR data. These models are formulated for 8 categories: ≤ −7, −6, …, 0. The lowest category is now the category ≤ −7, allowing the model to distinguish between the two types of -6 values (as mentioned before). The highest category is category 0, as no higher values have been observed, and as the value 0 has a unique interpretation across all years, regardless of the upper bounds 3 and 5 for the experimental ranges.

Summary goodness-of-fit statistic for the fitted models are given in Table 2. This table is designed as follows. The upper 4 (unnumbered) models are the standard type of multicategory logit models, without accounting for any varying experimental ranges. The models numbered 1 and 2 are again the only intercepts model (no time effect) and the baseline logit model, but both now accounting for the varying experimental ranges. These models however do not exploit the ordinal nature of the MIC categories *L* ≤ *j* ≤ *U* + 1. But both models do show a spectacular drop in AIC and BIC measures and clearly model 2 with time effects is the best fitting (but most complex) model.

All other models 3–15 are particular members of the new family, defined by Eq (7): models 3–10 represent models for which the effect of time is represented by one single slope, whereas models 11–15 contain two slopes (one for the left susceptible part and one for the right resistant part).

Within the group 3–10, model 6 and 7 have the lowest AIC and BIC. These models reflect a linear time effect at the resistant side of the MIC distribution and no or a sign effect at the susceptible side (a sign effect identical to the linear effect at the resistant side). Model 12, the best fitting model within the group 11–15, has linear effects with different slopes at both sides of the ECOFF. A closer inspection of the estimates of this model reveals however an issue with this model. Indeed the estimates for the slopes are $\hat{\beta_{s}}=-0.1171$ (se 0.0517) and $\hat{\beta_{r}}=0.1087$ (se 0.0249). The positive sign for $\hat{\beta_{r}}$ is to be expected, but the negative for $\hat{\beta_{s}}$ not. This negative slope implies that, with *J*_E_ = −4 (for the CIPR data) and *j* = −7, −6, −5

$$\frac{P(y=j|x+1)}{P(y=-4|x+1)}=e^{(-4-j)0.1171}\frac{P(y=j|x)}{P(y=-4|x)}$$

and

$$\frac{P(y=j+1|x+1)}{P(y=j|x+1)}=e^{-0.1171}\frac{P(y=j+1|x)}{P(y=j|x)}$$

reflecting, within the susceptible part of the MIC distribution, a shift to the left over time. Such shift is not happening in reality, and is an “artefact” of the changing experimental ranges over time. Fig 3 shows indeed that from 2008 onwards the experimental range stretches over two lower categories further. This increasing experimental range over time, the left censoring, and a possible time shift to the left of the susceptible part of the MIC distribution are confounded and cannot be disentangled. But by forcing both slopes to be positive, a possible shift to the left is excluded by the model formulation. Adding this (biologically justified) constraint was the motivation to add model 14 and 15. By excluding model 12, model 11 and 14 are, ex aequo, the best fitting models in this group of models with 8 parameters.

When looking across all 15 models, the baseline model 2 has the lowest AIC (1696.2), followed by model 12 (AIC 1699.6) and next by model 7 and 6 (AIC 1703.1 and 1703.7 respectively). According to BIC, model 7 and 6 are the best choice (BIC 1738.7 and 1739.3 respectively) followed by model 12 (BIC 1740.2) and model 8 (BIC 1742.1). Penalizing much more for complexity, BIC ranks model 2 as the worst. So AIC and BIC are completely in disagreement about model 2. Preferring more parsimonious models and excluding model 12, we select model 7 as best.

**Fig 4:**
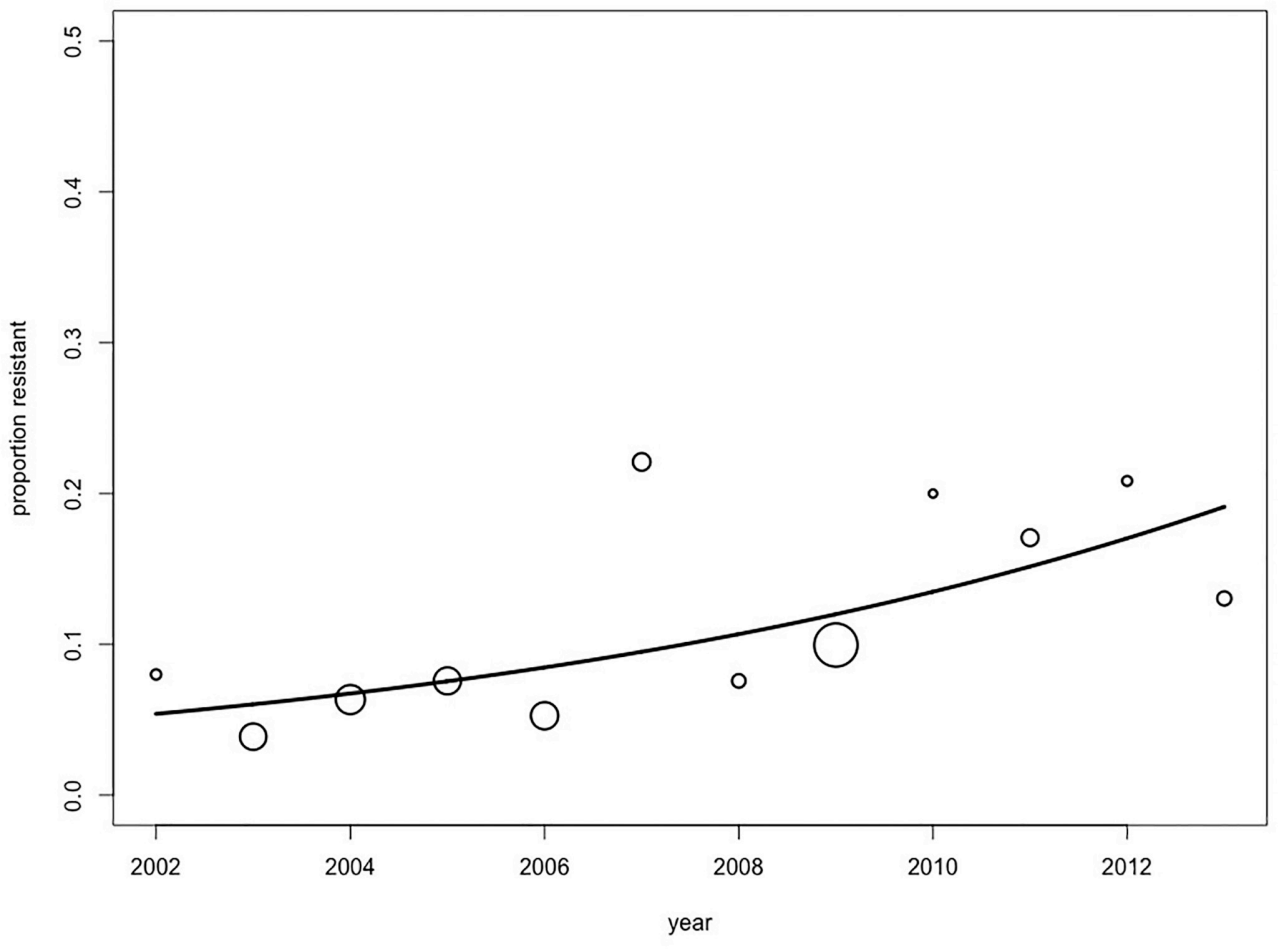
The CIPR data with fitted model. Scatterplot of observed proportions of resistant isolates by year, with bubble size proportional to the number of isolates. Solid line is the fitted model for the probability for an isolate to be resistant, as a function of time, and based on the final model 7.

Model 7 is nested in model 11 and 14. For both models 11 and 14, the slope *β*_*s*_ is not significantly different from 0 (p value 0.41 and 0.48 respectively), confirming the selection of model 7 as best model. Table 3 shows the estimates of intercepts and logit specific slopes, according to model 1, 2, 6, 7, 11 and 14 (best 2 in each group, excluding model 12) and Table 4 provides more details about the estimates of the best model 7. This model implies, with *J*_E_ = −4 and *j* = −7, −6, −5 that the odds *P*(*y* = *j*|*x*)/*P*(*y* = −4|*x*) of the categories on the susceptible part of the MIC distribution remain constant, and that for *j* = −3, −2, −1, 0, the odds

$$\frac{P(y=j|x+1)}{P(y=-4|x+1)}=e^{(j+4)0.059}\frac{P(y=j|x)}{P(y=-4|x)}$$

and

$$\frac{P(y=j+1|x+1)}{P(y=j|x+1)}=e^{0.059}\frac{P(y=j+1|x)}{P(y=j|x)}$$

reflect the increase to the neighboring higher category with about 6.1% with every additional year. Finally, we have that

$$\begin{array}{c} P\left(\text{isolate}\;\text{is}\;\text{resistant}|\text{time}\;x\right)=\frac{\sum_{j>-4}e^{\alpha_{j}+\left(j+4\right)0.059x}}{1+\sum_{j>-4}e^{\alpha_{j}+\left(j+4\right)0.059x}} \end{array}$$ (9)

which is an increasing function over time. Fig 4 shows the observed proportions of resistant isolates over time, together with the fitted model (9).

**Table 3:** The CIPR data: Estimates of intercepts and slopes of model 1, 2, 6, 7, 11 and 14.

	model 1		model 2		model 6		model 7		model 11		model 14	
cat	*α*	*β*	*α*	*β*	*α*	*β*	*α*	*β*	*α*	*β*	*α*	*β*
≤**-7**	-7.420	0	-7.428	-0.056	-7.427	-0.042	-7.430	0	-7.444	-0.017	-12.080	0.000
**-6**	1.186	0	0.050	0.139	1.523	-0.042	1.181	0	1.047	-0.017	1.182	0.000
**-5**	2.518	0	3.331	-0.099	2.856	-0.042	2.515	0	2.381	-0.017	2.515	0.000
**-4**	all 0 being the ECOFF category											
**-3**	-0.938	0	-0.177	-0.111	-1.051	0.042	-1.247	0.059	-1.324	0.065	-1.247	0.059
**-2**	-0.247	0	-0.777	0.122	-0.605	0.084	-0.892	0.118	-0.998	0.130	-0.892	0.118
**-1**	-0.471	0	-0.981	0.119	-1.093	0.126	-1.487	0.177	-1.634	0.196	-1.488	0.177
**0**	-2.363	0	-2.207	0.007	-3.269	0.168	-3.788	0.236	-3.987	0.261	-3.791	0.236

**Table 4:** The CIPR data. Model 7: estimate, standard error, t-value and p-value.

	est	se	est/se	p-value
***α*_−7_**	-7.430	10.276	-0.723	0.235
***α*_−6_**	1.181	0.220	5.367	0.000
***α*_−5_**	2.515	0.200	12.572	0.000
***α*_−4_**	0	0	ECOFF category	
***α*_−3_**	-1.247	0.284	-4.391	0.000
***α*_−2_**	-0.892	0.282	-3.159	0.001
***α*_−1_**	-1.487	0.354	-4.197	0.000
***α*_0_**	-3.788	0.581	-6.522	0.000
***β*_*r*_**	0.059	0.013	4.498	0.000

The lower panel of S1 Table shows the estimated probabilities based on model 7, and the middle part of Table 5 shows the expected counts. The lower part of Table 5 shows the expected counts again, but censored by the experimental ranges as in the original dataset. This facilitates the comparison between observed frequencies in the upper part of that same table. Overall, the observed and expected counts can be considered as close, given also that the counts for many categories are quite low. For 2012 the differences are larger, but, as also can be seen from Fig 3, the counts for the categories -6 and -5 are quite disproportionate as compared to those of the other years in the period 2008–2013.

**Table 5:** The CIPR data. The upper table: observed counts for each year. Middle table: based on the best fitting model 7, the expected counts for each year. Lower table: based on the best fitting model 7, the expected counts for each year, according to the observed experimental ranges.

**observed counts**									
**year**	≤**-7**	**-6**	**-5**	**ECOFF = -4**	**-3**	**-2**	**-1**	**0**	**total**
**2002**	46				3	1	0	0	50
**2003**	124				2	3	0	0	129
**2004**	133				2	2	5	0	142
**2005**	122				5	2	3	0	132
**2006**	126				1	4	2	0	133
**2007**	67				3	4	8	4	86
**2008**	0	13	44	4	0	3	1	1	66
**2009**	0	23	154	13	5	11	4	1	211
**2010**	0	7	24	1	1	5	2	0	40
**2011**	0	9	57	2	2	6	6	0	82
**2012**	21		15	2	0	5	5	0	48
**2013**	15		40	5	1	4	4	0	69
**total**	1067				25	50	40	6	1188
**expected counts**									
**year**	≤**-7**	**-6**	**-5**	**ECOFF = -4**	**-3**	**-2**	**-1**	**0**	**total**
**2002**	0	9	35	3	1	1	1	0	50
**2003**	0	24	90	7	2	3	2	0	128
**2004**	0	26	99	8	3	4	3	0	143
**2005**	0	24	91	7	3	4	3	0	132
**2006**	0	24	91	7	3	5	3	0	133
**2007**	0	15	58	5	2	3	3	0	86
**2008**	0	12	44	4	1	3	2	0	66
**2009**	0	36	138	11	5	10	9	1	210
**2010**	0	7	26	2	1	2	2	0	40
**2011**	0	14	52	4	2	5	5	1	83
**2012**	0	8	30	2	1	3	3	1	48
**2013**	0	11	42	3	2	5	5	1	69
**total**	0	210	796	63	26	48	41	4	1188
**expected counts accounting for the observed experimental ranges**									
**year**	≤**-7**	**-6**	**-5**	**ECOFF = -4**	**-3**	**-2**	**-1**	**0**	**total**
**2002**	47				1	1	1	0	50
**2003**	121				2	3	2	0	128
**2004**	133				3	4	3	0	143
**2005**	122				3	4	3	0	132
**2006**	122				3	5	3	0	133
**2007**	78				2	3	3	0	86
**2008**	0	12	44	4	1	3	2	0	66
**2009**	0	36	138	11	5	10	9	1	210
**2010**	0	7	26	2	1	2	2	0	40
**2011**	0	14	52	4	2	5	5	1	83
**2012**	8		30	2	1	3	3	1	48
**2013**	11		42	3	2	5	5	1	69
**total**	1069				26	48	41	4	1188

### The ISU VDL data

Swine samples from the Iowa State University Veterinary Diagnostic Laboratory (ISU VDL data) included a subset of data on *Salmonella* enterica I,4, [5],12:i:- tested with ceftiofur (TIO) from swine submissions. As in Zhang *et al*. [11], focus is on data obtained between 2011 and 2017. The upper part of Table 6 shows the observed counts in each MIC category for each year. Note that the experimental range does not change over time. For more details about this dataset, see Zhang *et al*. [11]. Using a hierarchical Bayesian latent class mixture model approach, Zhang *et al*. [11] detected a significantly increasing pattern in the non-resistant means for Salmonella I,4, [5],12:i:- tested with TIO.

**Table 6:** The ISU VDL data. The upper table: observed counts for each year. Lower table: based on the best fitting model 7, the expected counts for each year, given the observed total for each year.

**observed counts**								
**year**	≤ **-2**	**-1**	**0**	**ECOFF = 1**	**2**	**3**	> **3**	**total**
**2011**	3	7	2	0	0	2	2	16
**2012**	27	25	26	2	0	4	13	97
**2013**	35	35	90	15	1	9	30	215
**2014**	31	31	90	13	1	10	27	203
**2015**	23	40	109	11	2	28	38	251
**2016**	59	93	253	26	4	54	83	572
**2017**	22	45	258	25	2	42	48	442
**total**	200	276	828	92	10	149	241	1796
**expected counts**								
**year**	≤ **-2**	**-1**	**0**	**ECOFF = 1**	**2**	**3**	> **3**	**total**
**2011**	5	4	5	0	0	1	1	16
**2012**	24	20	33	4	0	6	10	97
**2013**	41	41	80	10	1	16	26	215
**2014**	29	35	82	11	1	17	28	203
**2015**	27	39	111	14	2	22	36	251
**2016**	46	81	279	31	3	50	81	572
**2017**	27	57	239	22	2	36	58	442
**total**	200	276	828	92	10	149	241	1796

Table 7 shows the goodness of fit statistics of the 10 fitted models. The classical baseline logit model with 12 parameters has AIC = 5498.9 and BIC = 5564.8. Note that AIC and BIC largely agree in ranking the 10 models. The best fitting model is model 7, with:

$$\begin{array}{c} f_{j}\left(x\right)=\{\begin{array}{cc} \left(j-J_{\text{E}}\right)\beta_{s}^{1}x+\text{sign}\left(j-J_{\text{E}}\right)\beta_{s}^{s}x^{2} & \text{for}\;\;\;L\leq j\leq J_{\text{E}},\\ 0 & \text{for}\;\;\;J_{\text{E}}\leq j\leq U+1, \end{array} \end{array}$$ (10)

reflecting a linear and quadratic trend on the “susceptible component” (below threshold) and no trend on the “resistant component” (above threshold). The linear effect magnifies for categories further away from the threshold category (factor (*j* − *J*_E_)), on the left end of the range, while the quadratic effect remains constant (sign(*j* − *J*_E_) = −1).

**Table 7:** The ISU VDL data: Summary goodness-of-fitted of all fitted model.

model	-2 log-likelihood	# par	AIC	BIC
**1: *f*_*s*_(*x*) = *x*, *f*_*r*_(*x*) = 0**	5509.2	7	5523.2(6)	5561.6(6)
**2: *f*_*s*_(*x*) = sign(*x*), *f*_*r*_(*x*) = 0**	5552.9	7	5566.9(8)	5605.3(8)
**3: *f*_*s*_(*x*) = sign(*x*), *f*_*r*_(*x*) = *x*, *β*_*s*_ ≠ *β*_*r*_**	5552.3	8	5568.3(9)	5612.2(9)
**4: *f*_*s*_(*x*) = *x*, *f*_*r*_(*x*) = sign(*x*), *β*_*s*_ ≠ *β*_*r*_**	5494.9	8	5510.9(5)	5554.8(4)
**5: *f*_*s*_(*x*) = *f*_*r*_(*x*) = *x*, *β*_*s*_ ≠ *β*_*r*_**	5490.3	8	5506.3(3)	5550.2(3)
**6: *f*_*s*_(*x*) = *f*_*r*_(*x*) = sign(*x*), *β*_*s*_ ≠ *β*_*r*_**	5552.8	8	5568.8(10)	5612.7(10)
**7: *f*_*s*_(*x*) = (*x*, sign(*x*)*x*^2^), *f*_*r*_(*x*) = 0**	5481.8	8	5497.8(1)	5541.8(1)
**8: *f*_*s*_(*x*) = (*x*, *x*^2^), *f*_*r*_(*x*) = 0**	5507.8	8	5523.8(7)	5567.8(7)
**9**: $f_{s}\left(x\right)=\left(x,\text{sign}\left(x\right)x^{2}\right),f_{r}\left(x\right)=x,\left\{\beta_{s}^{1},\beta_{s}^{2}\right\}\neq \beta_{r}$	5481.2	9	5499.2(2)	5548.6(2)
**10**: $f_{s}\left(x\right)=\left(x,x^{2}\right),f_{r}\left(x\right)=x,\left\{\beta_{s}^{1},\beta_{s}^{2}\right\}\neq \beta_{r}$	5490.1	9	5508.1(4)	5557.5(5)

**Fig 5:**
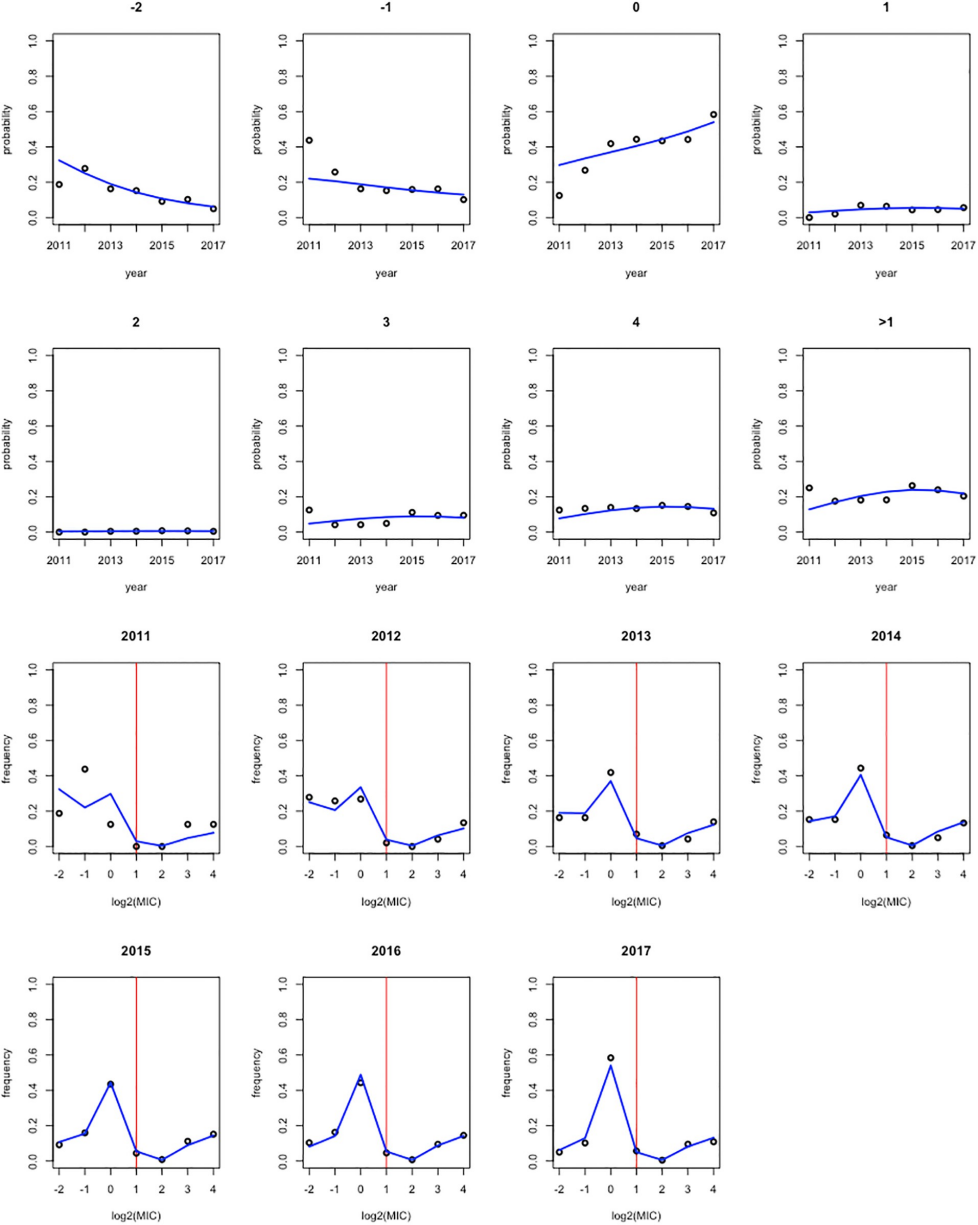
The ISU VDL data. Upper 2 rows: the observed and fitted trend over time using the best fitting model 7, for the probability for each of the 7 MIC values (on log2-scale), and, in the right lower panel, for the proportion resistant > 1 (1 being the threshold). Lower 2 rows: the observed and fitted MIC distribution using the best fitting model 7, for each year. The vertical red line refers to the threshold category.

S2 Table shows the fitted MIC distribution over the period 2011–2017. The observed and expected counts are shown in Table 6. The observed and expected frequencies for each MIC value, as a function of time is shown in the upper rows of Fig 5, and the observed and expected MIC distribution for each year is shown in the lower rows of Fig 5. The observed and expected frequencies are quite close, except for 2011. But the sample size of 2011 was very low, and much less than for the other years.

**Fig 6:**
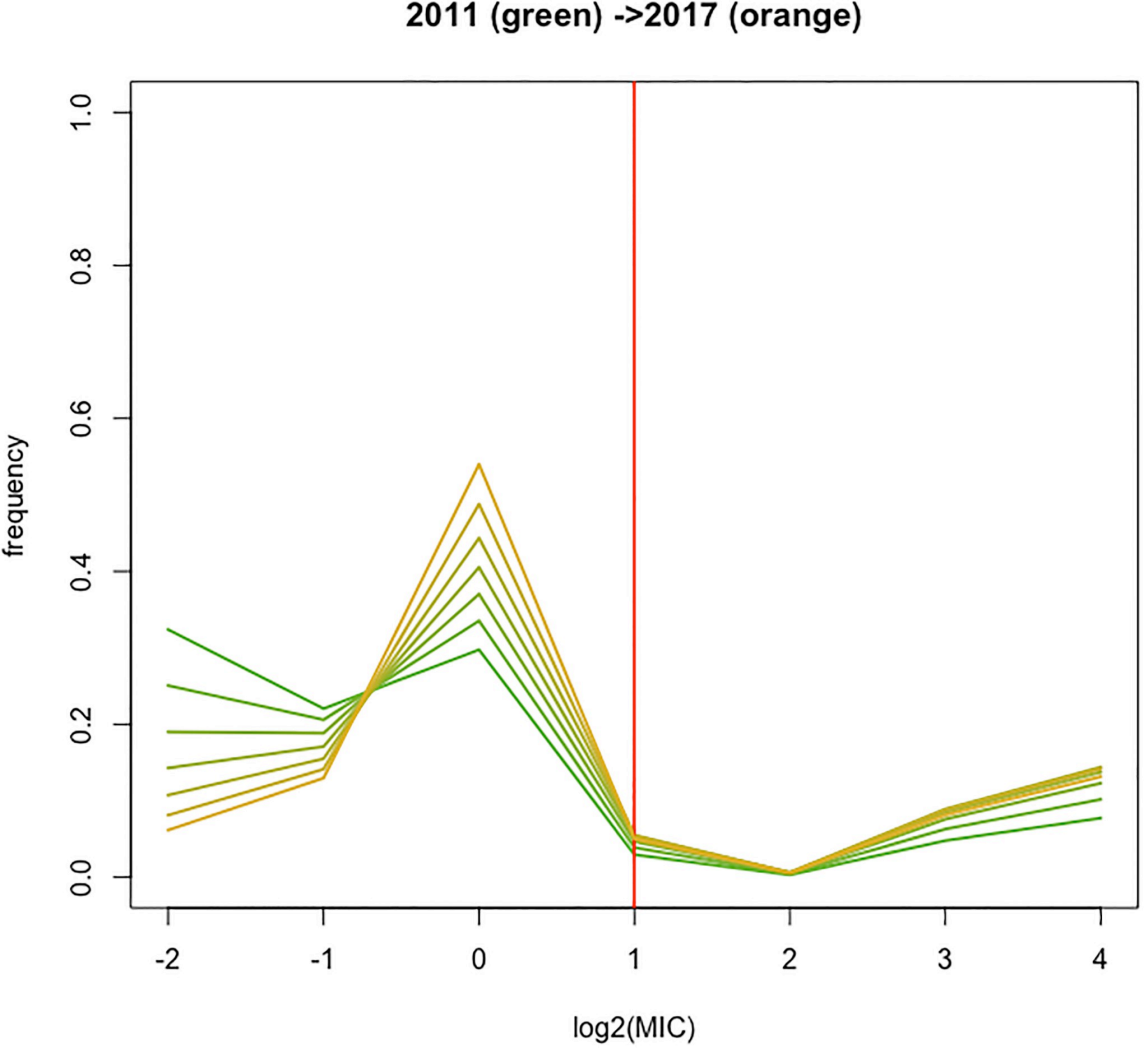
MIC distribution for the ISU VDL data. The observed and fitted MIC distribution, using the best fitting model 7, for each year. The vertical red line refers to the threshold category.

Table 8 shows the estimates for the intercepts and slopes of the best fitting model 7. The linear and quadratic effect shift probability mass from the left to the right categories situated at the left of the threshold category. Fig 6 shows this trend visually, with the green curve referring to year 2011, coloring gradually more yellow/orange to year 2017. The major trend happens in categories -2 (MIC ≤ 0.25) and category 0 (0 < MIC ≤ 1). This is in agreement with the conclusion in Zhang *et al*. [11], who “detected a significantly increasing pattern in the non-resistant means for *Salmonella* I,4, [5],12:i:- tested with TIO”.

**Table 8:** The ISU VDL data: Parameter estimates of best fitting model 7.

parameter	est	se	est/se	p-val
**intercept *α*_−2_**	2.39541	0.23003	10.41353	0.00000
**intercept *α*_−1_**	2.01123	0.18121	11.09873	0.00000
**intercept *α*_0_**	2.31049	0.14770	15.64328	0.00000
**intercept *α*_1_**	≡ 0	ECOFF category		
**intercept *α*_2_**	-2.20436	0.33104	-6.65886	0.00000
**intercept *α*_3_**	0.48417	0.13266	3.64975	0.00013
**intercept *α*_4_**	0.96445	0.12264	7.86438	0.00000
**slope** $\beta_{s}^{1}$	0.18787	0.02221	8.45848	0.00000
**slope** $\beta_{s}^{2}$	-0.03314	0.00639	-5.18241	0.00000

## Conclusion and discussion

We proposed the use of a new multicategory logit model for estimating and modelling the discrete MIC distributions over time. Not modelling the underlying continuous distribution avoids any assumption and inherently related misspecification of such distribution. Using censored data techniques, the approach also deals with varying experimental ranges. This approach allows the identification of trends above or below a breakpoint (here the ECOFF) as opposed to just changes in the proportion of wild/non-wild type strains. For instance, the estimated 6.1% increase to the neighboring category above the ECOFF with every additional year observed in the CIPR data may be suggestive of the accumulation of certain resistance mechanisms (e.g., specific mutations in *gyrA* and *parC*, and/or presence of plasmid-mediated quinolone-resistance genes) leading to decreased ciprofloxacin resistance and eventually high-level resistance [17].

The use of an ECOFF plays a central role in the construction of the model. This can be considered a strength as well as a weakness. It allows the identification of different time trends in the wild type and resistant subpopulation. But the method requires the existence of a well-defined ECOFF. Therefore, the proposed method is less suitable if such ECOFF is still “under discussion”, and obviously not suitable for the confirmation or determination of an ECOFF.

Interesting extension of the categorical model includes its generalization to more than two subpopulations, its extension to the bi- and multivariate setting. Also, the extension to hierarchical data, for which the inclusion of lab- or assay specific random effects can be included in the model, is an interesting topic for further research.

## Supporting information

S1 Fig The CIPR data. Barplots with fitted probabilities according to model 7, for each year in the period 2002–2013, but censored by the varying experimental ranges as in the original dataset, with barwidth proportional to the sample size of the respective year. The dotted vertical lines indicate: the smallest lower bound -7 and the highest upper bound 5 across all years (in red); the lower and upper bound of the experiments of the correspinding year (in green), the ECOFF (in black).
(PDF)

S1 Table The CIPR data. Estimated probabilities for each category, for each year, based on the standard baseline logit model (upper part) and the best model 7 (lower part).
(PDF)

S2 Table The ISU VDL data. Observed (upper panel) and estimated probabilities (lower panel) for each category, by year, based on the best fitting model 7.
(PDF)
