# Supplementary material for: A multicategory logit model detecting temporal changes in antimicrobial resistance
Source: PLoS One. 2022 Dec 1;17(12):e0277866. doi: 10.1371/journal.pone.0277866 (PMC9714861; doi:10.1371/journal.pone.0277866)

S2 The CIPR data. Barplots with fitted probabilities according to model 7, for each year in the period 2002-2013, but censored by the varying experimental ranges as in the original dataset, with barwidth proportional to the sample size of the respective year. The dotted vertical lines indicate: the smallest lower bound -7 and the highest upper bound 5 across all years (in red); the lower and upper bound of the experiments of the corresspinding year (in green), the ECOFF (in black).

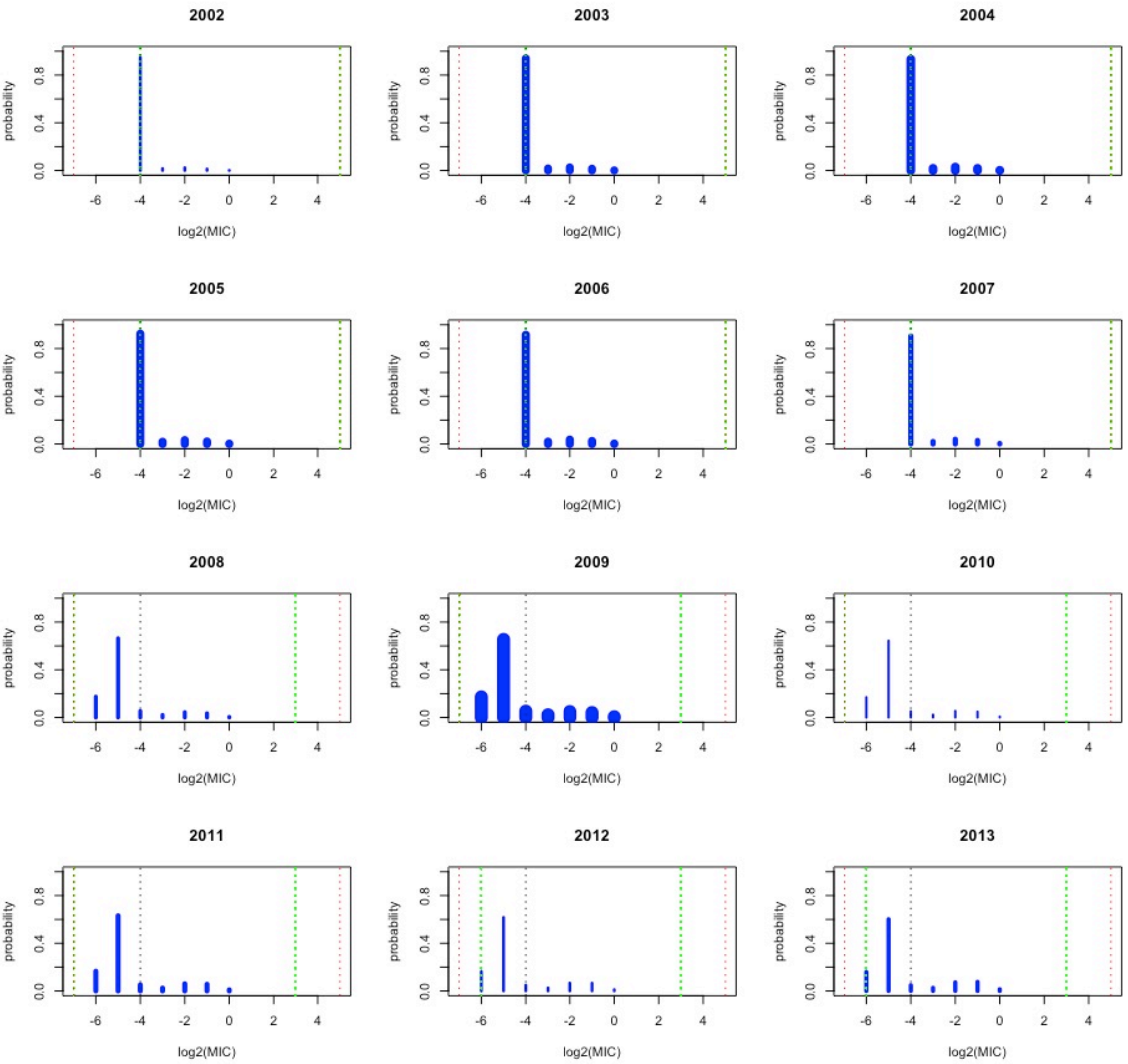

Supplement: S1 Fig — Barplots with fitted probabilities according to model 7, for each year in the period 2002–2013, but censored by the varying experimental ranges as in the original dataset, with barwidth proportional to the sample size of the respective year. The dotted vertical lines indicate: the smallest lower bound -7 and the highest upper bound 5 across all years (in red); the lower and upper bound of the experiments of the correspinding year (in green), the ECOFF (in black). (PDF) [file pone.0277866.s001.pdf]
