# Supplementary material for: A multicategory logit model detecting temporal changes in antimicrobial resistance
Source: PLoS One. 2022 Dec 1;17(12):e0277866. doi: 10.1371/journal.pone.0277866 (PMC9714861; doi:10.1371/journal.pone.0277866)
Supplement: S1 Table — Estimated probabilities for each category, for each year, based on the standard baseline logit model (upper part) and the best model 7 (lower part). (PDF) [file pone.0277866.s002.pdf]

Supporting information

S1 The CIPR data. Estimated probabilities for each category, for each year, based on the standard baseline logit model (upper part) and the best model 7 (lower part).

**standard baseline logit model**								
**year**	**≤ -7**	**-6**	**-5**	**ECOFF=-4**	**-3**	**-2**	**-1**	**0**
**2002**	— 0.000 —		0.001	0.988	0.009	0.001	0.001	0.001
**2003**	— 0.000 —		0.003	0.978	0.013	0.003	0.002	0.001
**2004**	— 0.001 —		0.008	0.958	0.019	0.007	0.005	0.002
**2005**	— 0.003 —		0.027	0.910	0.027	0.015	0.013	0.004
**2006**	— 0.010 —		0.081	0.803	0.037	0.034	0.027	0.007
**2007**	— 0.031 —		0.205	0.599	0.042	0.063	0.050	0.010
**2008**	— 0.071 —		0.391	0.335	0.035	0.087	0.070	0.011
**2009**	— 0.118 —		0.551	0.139	0.022	0.090	0.072	0.008
**2010**	— 0.162 —		0.637	0.047	0.012	0.076	0.061	0.005
**2011**	— 0.202 —		0.670	0.015	0.005	0.058	0.046	0.003
**2012**	— 0.241 —		0.674	0.004	0.002	0.043	0.034	0.002
**2013**	— 0.280 —		0.662	0.001	0.001	0.031	0.024	0.001
**best model 7**								
**year**	**≤ -7**	**-6**	**-5**	**ECOFF=-4**	**-3**	**-2**	**-1**	**0**
**2002**	3.4*	0.185	0.704	0.057	0.016	0.023	0.013	0.001
**2003**	3.4*	0.184	0.699	0.057	0.017	0.026	0.015	0.002
**2004**	3.3*	0.183	0.694	0.056	0.018	0.029	0.018	0.002
**2005**	3.3*	0.181	0.688	0.056	0.019	0.033	0.021	0.003
**2006**	3.3*	0.179	0.681	0.055	0.020	0.036	0.025	0.003
**2007**	3.2*	0.177	0.673	0.055	0.021	0.040	0.030	0.004
**2008**	3.2*	0.175	0.664	0.054	0.022	0.045	0.035	0.005
**2009**	3.1*	0.172	0.655	0.053	0.023	0.050	0.041	0.006
**2010**	3.1*	0.169	0.643	0.052	0.024	0.055	0.048	0.008
**2011**	3.0*	0.166	0.631	0.051	0.025	0.060	0.057	0.010
**2012**	3.0*	0.163	0.617	0.050	0.026	0.067	0.066	0.012
**2013**	2.9*	0.158	0.602	0.049	0.027	0.073	0.077	0.015

* in units of 10^{-5}
