# Supplementary material for: A multicategory logit model detecting temporal changes in antimicrobial resistance
Source: PLoS One. 2022 Dec 1;17(12):e0277866. doi: 10.1371/journal.pone.0277866 (PMC9714861; doi:10.1371/journal.pone.0277866)
Supplement: S2 Table — Observed (upper panel) and estimated probabilities (lower panel) for each category, by year, based on the best fitting model 7. (PDF) [file pone.0277866.s003.pdf]

S3 The ISU VDL data. Observed (upper panel) and estimated probabilities (lower panel) for each category, by year, based on the best fitting model 7.

**year**	**$\leq$ -2**	**-1**	**0**	**ECOFF=1**	**2**	**3**	**>3**
**2011**	0.188	0.438	0.125	0.000	0.000	0.125	0.125
**2012**	0.278	0.258	0.268	0.021	0.000	0.041	0.134
**2013**	0.163	0.163	0.419	0.070	0.005	0.042	0.140
**2014**	0.153	0.153	0.443	0.064	0.005	0.049	0.133
**2015**	0.092	0.159	0.434	0.044	0.008	0.112	0.151
**2016**	0.103	0.163	0.442	0.045	0.007	0.094	0.145
**2017**	0.050	0.102	0.584	0.057	0.005	0.095	0.109

**year**	**$\leq$ -2**	**-1**	**0**	**ECOFF=1**	**2**	**3**	**>3**
**2011**	0.324	0.221	0.297	0.030	0.003	0.048	0.077
**2012**	0.251	0.206	0.335	0.039	0.004	0.063	0.102
**2013**	0.190	0.189	0.370	0.047	0.005	0.076	0.123
**2014**	0.143	0.171	0.405	0.052	0.006	0.085	0.138
**2015**	0.107	0.155	0.443	0.055	0.006	0.089	0.144
**2016**	0.081	0.141	0.488	0.054	0.006	0.088	0.142
**2017**	0.062	0.130	0.540	0.050	0.006	0.081	0.132
